# Body size and tube voltage dependent corrections for Hounsfield Unit in medical X-ray computed tomography: theory and experiments

**DOI:** 10.1038/s41598-020-72707-y

**Published:** 2020-09-24

**Authors:** Xiaoming Zheng, Yazan Al-Hayek, Chris Cummins, Xiaotian Li, Laura Nardi, Khaled Albari, James Evans, Evan Roworth, Ty Seaton

**Affiliations:** 1 Medical Radiation Sciences, School of Dentistry and Health Sciences, Faculty of Science, Charles Sturt University, Wagga Wagga, NSW 2678 Australia; 2 Changchun Institute of Optics, Fine Mechanics and Physics, Chinese Academy of Sciences, Changchun, 130033 Jilin China; 3 I-Med Regional Imaging Riverina, 36 Hardy Avenue, Wagga Wagga, NSW 2650 Australia; 4 Afia Radiotherapy and Nuclear Medicine Centre, Ibn Khadoun Street, Amman, 11183 Jordan; 5 The X-Ray Group Pty Ltd, 9 Stanley Street, Wodonga, VIC 3690 Australia

**Keywords:** Biophysics, Medical research, Engineering, Physics

## Abstract

The purpose of this work is to present a body size and tube voltage dependent correction scheme for the Hounsfield Unit, HU, in medical X-ray Computed Tomography imaging. Boltzmann photon transport equation was employed to study X-ray interaction with bulk water in CT imaging. Experimentally measured X-ray output in body of phantoms and attenuation cross sections of water were employed in the derivation of beam intensity in X-ray imaging. A Somatom Emotion CT scanner from Siemens and electron density phantoms from CIRS were employed to acquire CT images of different body sizes and different tissue materials located at different depths from body’s surface. Tube voltage and depth dependent effective attenuation of bulk water was found from theoretical analysis in agreement with measured size-specific correction factors for CTDI_vol_ under different tube voltages. A size and tube voltage dependent correction scheme for the Hounsfield Unit is established. For the same tissue material, body size has much larger impact on the CT number variations than that of depth from the body surface in phantom measurements. Good results were achieved by applying the established correction scheme on the experimentally measured CT number variations under different tube voltages and body sizes.

## Introduction

Accurate image numbers in X-ray computed tomography are critical not only for quantitative diagnostic imaging^1,2^ but also for dose calculations in radiotherapy^3,4^. A number of factors may contribute to the inaccuracy of CT numbers such as CT image’s inherent noise^5,6^, various artefacts such as beam hardening and metal artefact, and various calibration or correction schemes embedded in image reconstruction algorithms^1^. In addition to afore mentioned major sources for CT number inaccuracy, CT numbers were also found to be body size dependent^1,3^. The variations of CT numbers on body size was attributed to beam hardening^1^ or CT system’s calibration^3^. The beam hardening is well known in CT imaging as polychromatic X-rays are employed in clinical X-ray imaging^1^. Various beam hardening correction schemes are implemented in current clinical scanners although the exact correction scheme of a specific CT scanner is manufacturer’s proprietary information. It appears that the beam hardening correction is calibrated only with a specific body size and tube voltage, kVp, because CT numbers will still change if the body size is varied^3^. A system calibration or correction scheme for all body sizes would be required in order to remove the size dependent variations of CT numbers.

In X-ray computed tomography, image numbers are in Hounsfield Unit which are calculated by using attenuation co-efficient of water as the reference^1^. The interaction of X-rays with bulk water may assist with the understanding of body size effect upon CT numbers. The purpose of this work is to present a theoretical analysis on the X-ray interactions with bulk water employing the Boltzmann photon transport equation. A body size and kVp dependent correction factor is then established. CT images of different phantom sizes under various kVps were acquired to test the proposed correction scheme.

## Materials and methods

### Boltzmann photon transport equation

Time independent X-ray photon transport equation can be expressed as^7^:

1 $${\varvec{\omega}} \cdot \nabla {\varvec{f}}\left( {{\varvec{r}},{\varvec{\omega}},\lambda } \right) = - \mu \left( {\varvec{\lambda}} \right){\varvec{f}}\left( {{\varvec{r}},{\varvec{\omega}},\lambda } \right) + \mathop \smallint \limits_{0}^{\infty } {\varvec{d}}\lambda^{\prime}\mathop \smallint \limits_{0}^{{4{\varvec{\pi}}}} {d\omega^{\prime}}K\left( {{{\varvec{\upomega}}},{{\varvec{\uplambda}}},\user2{\omega^{\prime}},\lambda^{\prime}} \right){\varvec{f}}\left( {{\varvec{r}},\user2{\omega^{\prime}},\lambda^{\prime}} \right) + \hat{J}\left( {{\mathbf{r}},{\varvec{\omega}},{\varvec{\lambda}}} \right)$$

where:

${\varvec{f}}\left( {{\varvec{r}},{\varvec{\omega}},\lambda } \right)$ is the X-ray photon’s flux of wavelength λ at spatial location **r**;

$\left( {{\varvec{\omega}},\lambda } \right)\user2{ and }\left( {\user2{\omega^{\prime}},\lambda^{\prime}} \right)$ are flowing-out and flowing-in photon’s momentum-space variables;

$\mu \left( {\varvec{\lambda}} \right)$ is the energy (wave length) dependent attenuation co-efficient;

**Ĵ(r,**${\varvec{\omega}},{\varvec{\lambda}})$ is the X-ray photon source at spatial location r and.

$\kappa \left( {{{\varvec{\upomega}}},{{\varvec{\uplambda}}},\user2{\omega^{\prime}},\lambda^{\prime}} \right)$ is the interaction kernel of X-ray photon with the matter.

For X-ray interaction with matter, $\kappa \left( {{{\varvec{\upomega}}},{{\varvec{\uplambda}}},\user2{\omega^{\prime}},\lambda^{\prime}} \right)$ can be expressed as^7^:

2 $$\kappa \left( {{{\varvec{\upomega}}},{{\varvec{\uplambda}}},\user2{\omega^{\prime}},\lambda^{\prime}} \right) = \kappa_{{\varvec{R}}} \left( {{{\varvec{\upomega}}},{{\varvec{\uplambda}}},\user2{\omega^{\prime}},\lambda^{\prime}} \right) + \kappa_{{\varvec{C}}} \left( {{{\varvec{\upomega}}},{{\varvec{\uplambda}}},\user2{\omega^{\prime}},\lambda^{\prime}} \right) + \mathop \sum \limits_{{\varvec{i}}} \kappa_{{{\varvec{P}}_{{{\varvec{\lambda}}_{{\varvec{i}}} }} }} \left( {{{\varvec{\upomega}}},{{\varvec{\uplambda}}},\user2{\omega^{\prime}},\lambda^{\prime}} \right)$$

where,

$\kappa _{{\varvec{R}}} \left( {{{\varvec{\upomega}}},{{\varvec{\uplambda}}},\user2{\omega^{\prime}},\lambda^{\prime}} \right)$ is Rayleigh interaction kernel;

$\kappa _{{\varvec{C}}} \left( {{{\varvec{\upomega}}},{{\varvec{\uplambda}}},\user2{\omega^{\prime}},\lambda^{\prime}} \right)$ is the Compton interaction kernel and.

$\mathop \sum \limits_{{\varvec{i}}} \kappa_{{{\varvec{P}}_{{{\varvec{\lambda}}_{{\varvec{i}}} }} }} \left( {{{\varvec{\upomega}}},{{\varvec{\uplambda}}},\user2{\omega^{\prime}},\lambda^{\prime}} \right)$ is the photoelectric interaction kernel; and

3 $$\mu = \sigma_{C} + \sigma_{R} + \tau$$

where ${\sigma }_{C} \ and \ {\sigma }_{R}$ are Compton and Rayleigh integral attenuation coefficients and $\tau$ is the photoelectric attenuation coefficient.

In computed tomography, X-ray photon transport can be considered as one-dimensional. The one-dimensional Boltzmann equation is given as^7^:

4 $$\eta \frac{{\partial {\varvec{f}}\left( {{\varvec{x}},{\varvec{\omega}},\lambda } \right)}}{{\partial {\varvec{x}}}} = - \mu \left( {\varvec{\lambda}} \right){\varvec{f}}\left( {{\varvec{x}},{\varvec{\omega}},\lambda } \right) + \mathop \smallint \limits_{0}^{\infty } {\varvec{d}}\lambda^{\prime}\mathop \smallint \limits_{0}^{{4{\varvec{\pi}}}} {d\omega^{\prime}}K\left( {{{\varvec{\upomega}}},{{\varvec{\uplambda}}},\user2{\omega^{\prime}},\lambda^{\prime}} \right)U\left( x \right){\varvec{f}}\left( {{\varvec{x}},\user2{\omega^{\prime}},\lambda^{\prime}} \right) + {\varvec{I}}_{0} {{\varvec{\updelta}}}\left( {\mathbf{x}} \right){{\varvec{\updelta}}}\left( {{{\varvec{\upomega}}} - {\varvec{\omega}}_{0} } \right){{\varvec{\updelta}}}\left( {{\varvec{\lambda}} - {\varvec{\lambda}}_{0} } \right)$$

where

$$\eta = \cos \omega_{x} = 1;$$

$$U\left( x \right) = \begin{array}{*{20}c} {1 \ for \ x > 0} \\ {0\ for \ x < 0} \\ \end{array}$$ and

$I_{0} {\updelta }\left( {\text{x}} \right){\updelta }\left( {{\upomega } - \omega_{0} } \right){\updelta }\left( {\lambda - \lambda_{0} } \right) = 0$ as there is no X-ray source in the body.

In computed tomography, X-rays are generated using an X-ray tube by applying a kilo-voltage, kVp. The X-ray photon energies range from 0 to a peak tube voltage Vp. X-ray photon flux is measured as exposure (dose) output, or, photon flux per unit area, i.e. intensity I. Considering X-ray photon flux per unit area and replace wavelength, λ, by voltage, V, Eq. (4) becomes:

5 $$\frac{{\partial {\varvec{I}}\left( {{\varvec{x}},{\varvec{\omega}},V} \right)}}{{\partial {\varvec{x}}}} = - \mu \left( V \right){\varvec{I}}\left( {{\varvec{x}},{\varvec{\omega}},V} \right) + \mathop \smallint \limits_{0}^{{{\varvec{V}}_{{\varvec{p}}} }} {\varvec{d}}V^{\prime}\mathop \smallint \limits_{0}^{{4{\varvec{\pi}}}}{d\omega^{\prime}}K\left( {{{\varvec{\upomega}}},{\text{V}},\user2{\omega^{\prime}},V^{\prime}} \right)U\left( x \right){\varvec{I}}\left( {{\varvec{x}},\user2{\omega^{\prime}},V^{\prime}} \right)$$

**Figure 1:**
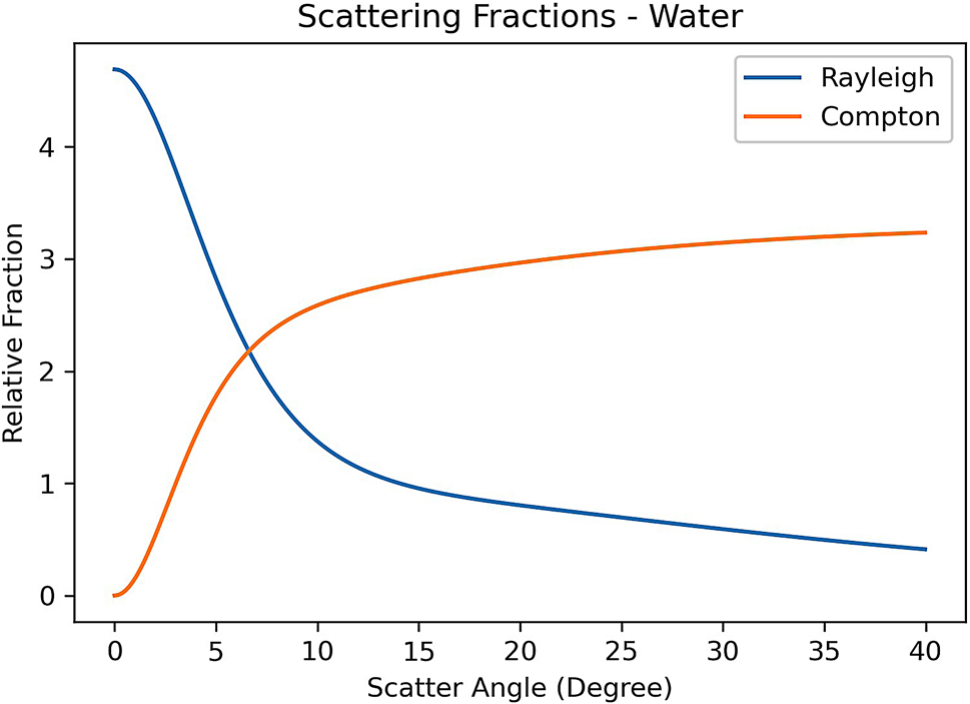
The relative scattering fractions of X-ray Rayleigh and Compton interactions with water deviated from the beam direction (x). The figure was generated using the SAP code^8^.

The three components of interaction kernel $\kappa \left( {{{\varvec{\upomega}}},{\text{V}},\user2{\omega^{\prime}},V^{\prime}} \right)$ can be considered separately. In computed tomography, those X-ray photons with ${\omega }_{x}$ or in x direction are detected. Most of the photoelectric interacted X-rays will be absorbed by the medium. Figure 1 shows the relative scattering fractions of Rayleigh and Compton interactions with water as a function of the scattering angles. The data was from Fernandez et al.^8^. CT detectors are located at zero scattering angle or perpendicular to the x direction.

Figure 1 suggests that the X-ray photons following Rayleigh interaction will be detected but not Compton scattering at zero angle. It is worth noting that multiple Compton scatterings will be detected at zero angle. However, these Compton photons detected are mainly image noise, not image signals. It follows that:

6 $$\kappa \left( {{{\varvec{\upomega}}},{\text{V}},\user2{\omega^{\prime}},V^{\prime}} \right) = \kappa_{{\varvec{R}}} \left( {{{\varvec{\upomega}}},{\text{V}},\user2{\omega^{\prime}},V^{\prime}} \right)$$

**Figure 2:**
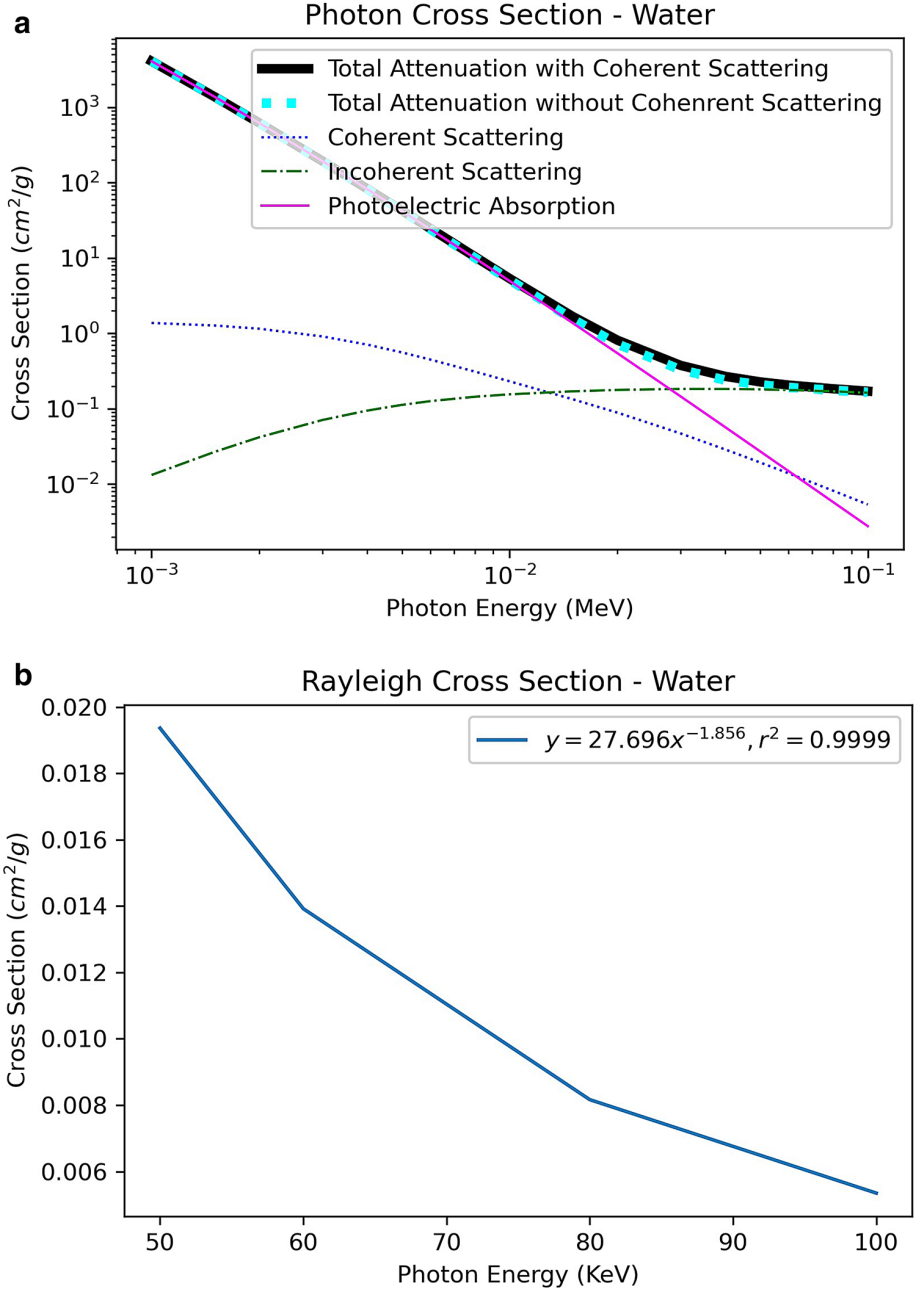
(**a**) Various photon cross section components of water. Data were generated from XCOM program^9^. (**b**) The plot of Rayleigh cross section from 50–100 keV of (**a**).

Figure 2a shows the various cross section components of X-ray interaction with water in the energy range from 0 to 100 keV. The data was from Berger et al.^9^. Figure 2b shows the Rayleigh component of the total cross section within X-ray imaging’s effective energy range of 50–100 keV from Fig. 2a.

Figure 2b suggests that within X-ray computed tomography energy range,

7 $$\mathop \smallint \limits_{0}^{{4{\varvec{\pi}}}} \user2{d\omega^{\prime}}\kappa\left( {{{\varvec{\upomega}}},{\text{V}},\user2{\omega^{\prime}},V^{\prime}} \right) = AV^{ - 1.856}$$

where A = 27.696 is a constant from the curve fitting of Fig. 2b.

The X-ray intensity (output) in phantom was measured experimentally^10^ and expressed as^11^:

8 $$I\left( {x,V} \right) = KA_{s} V^{\alpha x + \beta } e^{{ - \left( {\tau - \sigma \ln \left( V \right)} \right)x}} = BV^{{\left( {\alpha + \sigma } \right)x + \beta }} e^{ - \tau x} = BV^{\varepsilon x + \beta } e^{ - \tau x}$$

where K, B, $\alpha , \beta , \theta ,\varepsilon$ are constant: B is X-ray tube’s milli-ampere-second (mAs) dependent constant; $\varepsilon =\alpha +\sigma =0.034+0.0.023=0.057, \beta \approx 2 \ and \ \tau \approx 0.5302$ [^10,11^].

In CT imaging, the polychromatic X-rays can be represented by a single effective energy (voltage)^1,12^ although the exact effective energy is system dependent as different beam filtrations are employed for different imaging systems. For any voltage V, Eq. (5) now becomes:

9 $$\frac{{\partial \left( {BV^{{\varepsilon x + \beta }} e^{{ - \tau x}} } \right)}}{{\partial x}} = - \mu \left( V \right)\left( {BV^{{\varepsilon x + \beta }} e^{{ - \tau x}} } \right) + \mathop \smallint \limits_{0}^{V} dV{^\prime}AV^{{\prime}{ - 1.856}} \left( {BV^{{\prime}{\varepsilon x + \beta}} e^{{ - \tau x}} } \right)$$

Or

10 $$\left( {\varepsilon \ln \left( V \right) - \tau } \right)I\left( {x,V} \right) = - \mu \left( V \right)I\left( {x,V} \right) + \left( {\frac{{AV^{ - 0.856} }}{\varepsilon x + 1.144}} \right)I\left( {x,V} \right)$$

Or

11 $$\mu \left( V \right) = \tau - \varepsilon \ln \left( V \right) + \left( {\frac{{AV^{ - 0.856} }}{\varepsilon x + 1.144}} \right)$$

Equation (11) suggests that the water’s bulk effective attenuation is not only dependent on energy (V) but also on the depth from the entrance surface x. Let,

12 $$f\left( {x,V} \right) = \frac{{AV^{ - 0.856} }}{\varepsilon x + 1.144}$$

**Figure 3:**
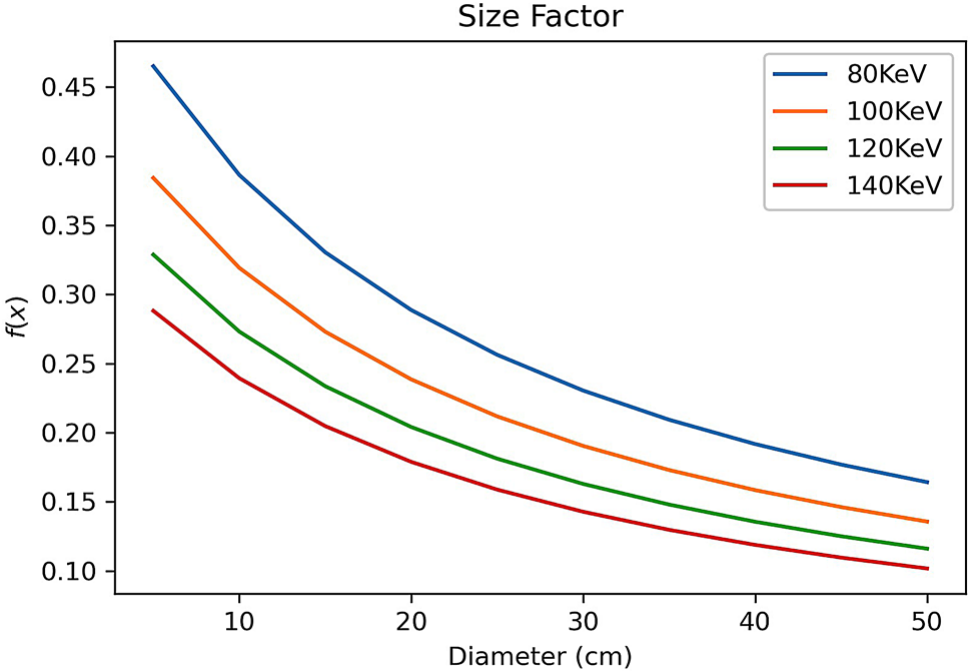
Size factor f(x,V) as a function of depth x or diameter of the body size in good agreement with Fig. 8.11a of reference^5^.

Figure 3 shows a plot of $f\left(x,V\right)$ vs x which is in good agreement with the measured kVp dependent size-specific correction factors for the absorbed dose CTDI_vol_^5^.

The term $f\left(x,V\right)$ suggests a depth dependent effective attenuation of bulk water and Fig. 3 suggests that $f\left(x,V\right)$ can be used to correct for size effect on the effective attenuation of bulk water. The term $f\left(x,V\right)$ is a result of Rayleigh scattering as shown in the previous mathematical analysis. It suggests a reduced effective attenuation as the depth from the entrance surface is increased or the body size is increased in X-ray CT imaging. The reduced effective attenuation is termed as beam hardening when polychromatic X-rays are passing through the bulk water, as the lower energy (soft) part of X-rays are removed from the spectrum. The above analysis shows that the Rayleigh scattering is a part of beam hardening effect in X-ray CT imaging where Rayleigh scattering adds elastic (coherent) scattering photons back to the beam intensity for imaging. In Monte Carlo simulations, X-ray photons are treated as particles which is the same as electrons and neutrons. The energy dependent mean free path length, ${x}_{0}\left(V\right),$ of Rayleigh scattering can be expressed as^13^:

13 $$x_{0} \left( V \right) = \frac{1}{{{\varvec{\sigma}}_{{\varvec{R}}} \left( {\varvec{V}} \right)}}$$

where ${{\varvec{\sigma}}}_{{\varvec{R}}}\left({\varvec{V}}\right)$ is the energy dependent cross section of Rayleigh scattering; Employing continue slowing down approximation and range straggling for X-ray photon particles^13^, a depth or body size dependent effective attenuation factor of water can be defined as:

14 $$f_{x} \left( {x,V} \right) = \frac{{AV^{ - 0.856} }}{{\varepsilon \left( {x_{0} \left( V \right) + x} \right) + 1.144}}$$

And an effective attenuation correction factor for bulk water can be calculated as:

15 $$f_{c} \left( {x,V} \right) = \frac{{AV^{ - 0.856} }}{{\varepsilon x_{0} \left( V \right) + 1.144}} - \frac{{AV^{ - 0.856} }}{{\varepsilon \left[ {(x_{0} \left( V \right) + x} \right] + 1.144}} = \Delta \mu \left( {x,V} \right)$$

where x is the depth from the entrance surface or body size r = d/2. In X-ray CT, image values are presented in Hounsfield Unit which takes water’s attenuation coefficient as their reference^1^:

16 $$HU = \left( {\frac{{\mu \left( V \right) - \mu_{w} \left( V \right)}}{{\mu_{w} \left( V \right)}}} \right) \times 1000$$

where $\mu \left(V\right)$ is energy dependent material’s/tissue’s attenuation coefficient and ${\mu }_{w}\left(V\right)$ is energy dependent water’s attenuation coefficient. In calculating the CT image values using Eq. (16), the effective attenuation of water should be employed:

17 $$HU\left( {x,V} \right) = \left( {\frac{{\mu \left( {x,V} \right) - \mu_{w} \left( V \right) + f_{c} \left( {x,V} \right)}}{{\mu_{w} \left( V \right) - f_{c} \left( {r,V} \right)}}} \right) \times 1000$$

where: ${f}_{c}\left(x,V\right)$ and ${f}_{c}\left(r,V\right)$ are depth and size dependent correction factors expressed in Eq. (15).

### Experimental measurements

**Figure 4:**
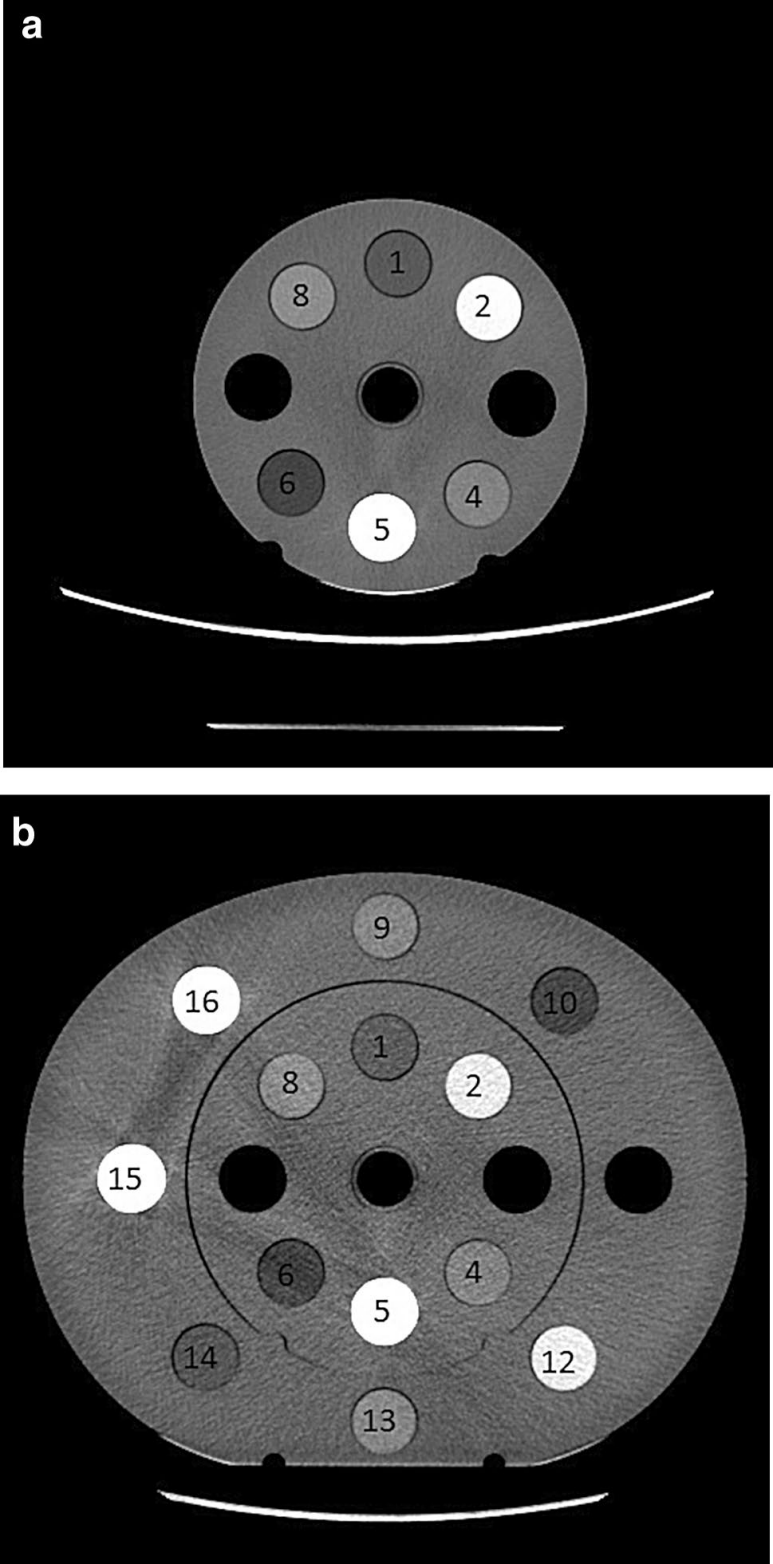
(**a**) Small electron density phantom with nine holes of 6 cm in diameter for inserting various tissue materials. (**b**) Large electron density phantom constructed by adding an elliptical ring disk to the small circular disk. There are total 17 holes of 6 cm in diameter for inserting various materials within this construct.

A Somatom Emotion CT scanner from Siemens and electron density phantoms from CIRS were employed in this study. The CIRS’s model 062 M electron density phantoms consist of 2 nested disks made from plastic water, 5 cm in thickness. These can be used to represent both head (small circular disk, 18 cm in diameter, as shown in Fig. 4a) and abdomen (a larger elliptical disk ring, 33 cm × 27 cm, is added to the small circular disk as shown in Fig. 4b) body parts. There are nine holes of 6 cm in diameter within the small circular disk and eight holes of 6 cm in diameter within the large elliptical disk ring for inserting different materials (see Fig. 4).

The first measurement was carried out by filling all the holes with water balloons and both head (Fig. 4a) and abdomen (Fig. 4b) were scanned under tube voltages of 80, 110 and 130 keV. Each of these scans was repeated three times. The second measurement was carried out by filling number 6 hole with tissue equivalent materials: breast, adipose, liver, muscle, bone200, bone800 and bone1250, in turn, with the rest holes filled with water balloons as in the first measurement. These tissue equivalent (non-human) materials’ inserts are provided by the CIRS as a part of the model 062 M electron density phantoms (not from any tissue bank). The use of these tissue equivalent materials’ inserts and experimental protocol were approved by the ethics committee of Riverina Cancer Care Centre. The head sized phantom (Fig. 4a) was scanned first and then followed by adding the outer disk ring (all holes of the ring disk were filled with water balloons) to make abdomen sized phantom (Fig. 4b). Both of the two sized phantoms were scanned three times each, under three kVps of 80, 110 and 130. Finally, the abdomen sized phantom was scanned by filling the same seven tissue materials at number 14 hole of the outer ring disk, in turn, with the rest holes filled with water balloons. Again, the scans were repeated three times under the three kVps of 80, 110 and 130 for each of the tissue material’s acquisitions. The system’s default exposure 280 mAs were used for all of the scans with routine helical abdomen protocol of a large bowtie filter (0.75 s gantry rotation time, 0.9 Pitch, 6 × 1.5 collimation and 50 cm FOV). The CT image values at holes number 6, 14 and the centre hole were calculated by employing the ImageJ software^14^. The tissue equivalent materials CT numbers were calculated by averaging eight central slices from each of the scans.

## Results

**Figure 5:**
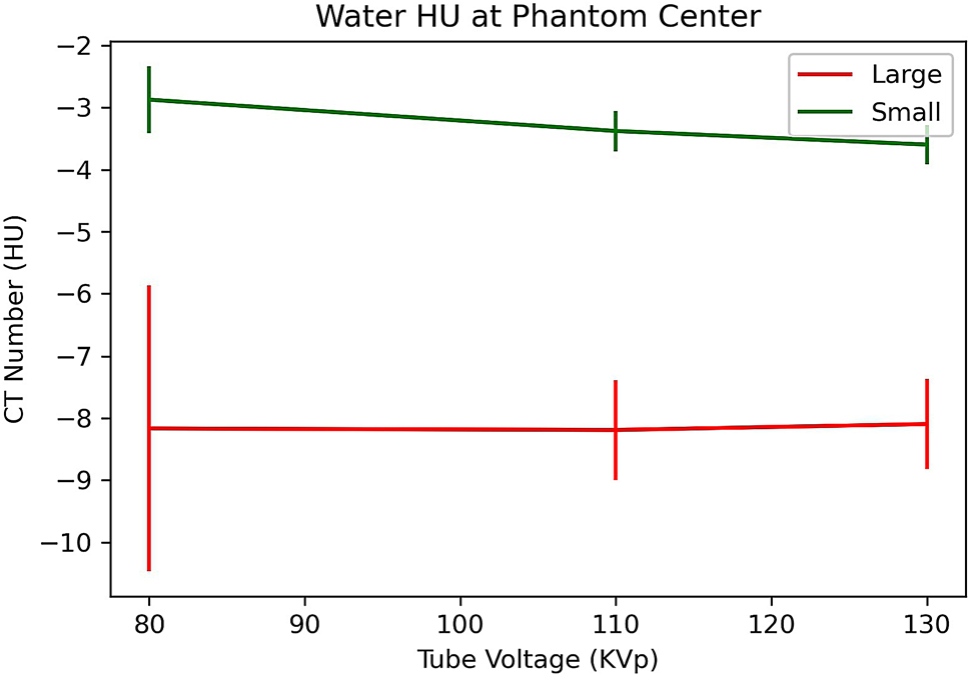
Measured CT numbers of water at the centre of the two sized phantoms (18 cm and 30 cm in diameter).

Figure 5 shows the measured CT numbers at the centre of the two sized phantoms. It shows that: (i) the water’s CT number is non-zero negative and decreased as the phantom size is increased; (ii) the CT numbers were constant for the large abdomen sized phantom (kVp independent). Water’s CT numbers should be zero according to the definition of the Hounsfield Unit for all kVps and body sizes. The constant water CT numbers suggest that the system was calibrated at the large phantom size of averaged 30 cm in diameter.

**Figure 6:**
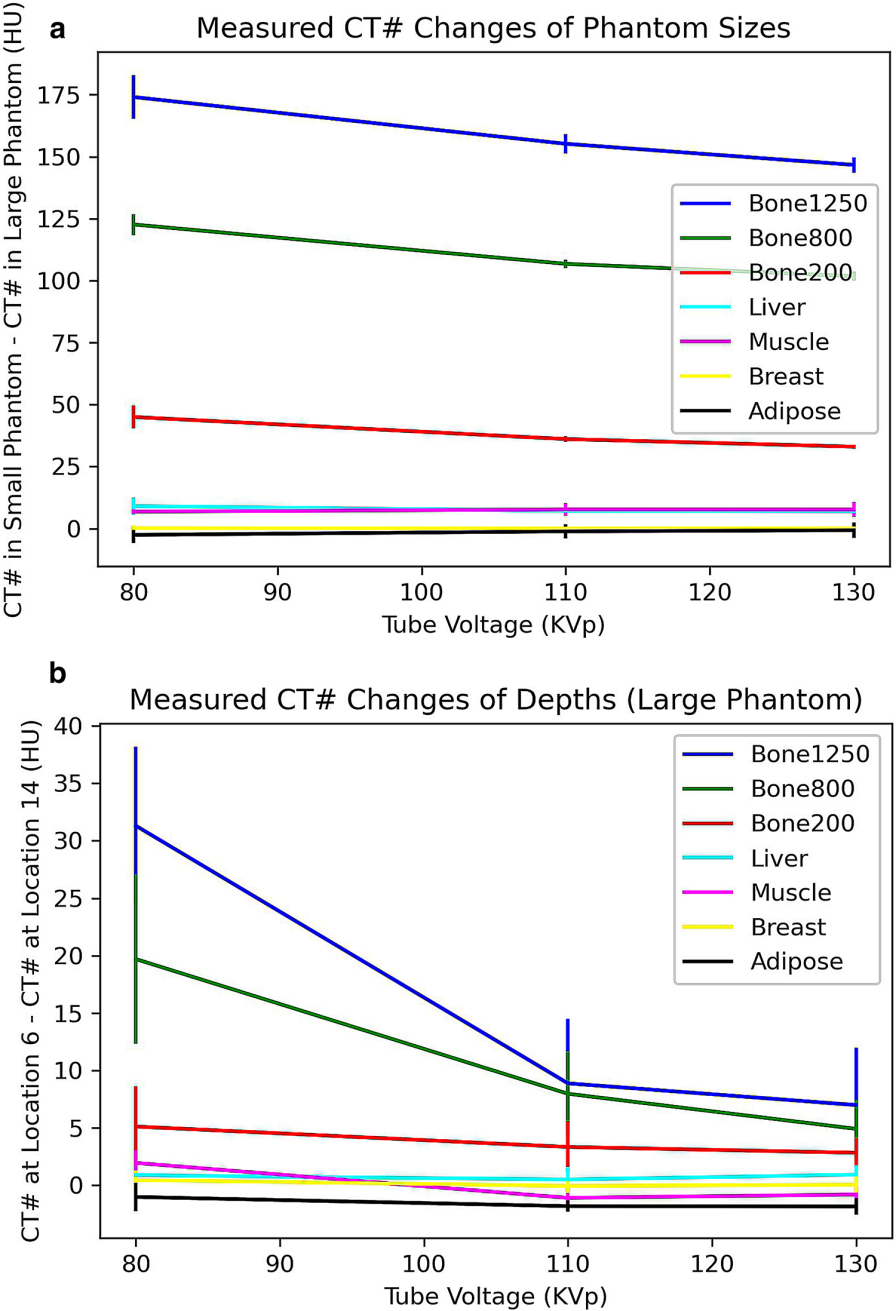
Measured CT number changes. (**a**) CT number changes at number 6 hole from small circular disk phantom to the large elliptical disk phantom; (**b**) CT number changes at from number 6 hole to number 14 hole within the same large elliptical disk phantom.

Figure 6 shows the measured CT number changes from the two phantom sizes (Fig. 6a) and two depth locations within the large phantom (Fig. 6b) under various tube voltages for the seven tissue equivalent materials. These are averaged values from the eight central slices of the three repeated measurements. Figure 6a shows the size and tube voltage effects on the CT numbers. The largest difference in CT number (174 HU) was observed for tissue equivalent material of Bone1250 under tube voltage of 80 keV. Figure 6b shows the phantom depth and tube voltage effects on the CT numbers (within the same large phantom). The largest difference in CT number (31 HU) was observed for the same tissue equivalent material, Bone1250, at 80 keV. It suggests that the body size has a much larger impact on the CT numbers than that of the depth from the entrance surface because the distance between hole 6 and hole 14 is approximately 6 cm and the body size radius difference is also approximately 6 cm. Figure 6b also shows that the curve shapes of CT number changes were depressed at tube voltage of 110 keV in contrast to the curve shapes from size effect in Fig. 6a. This suggests that the system was calibrated at the tube voltage of 110 keV.

**Figure 7:**
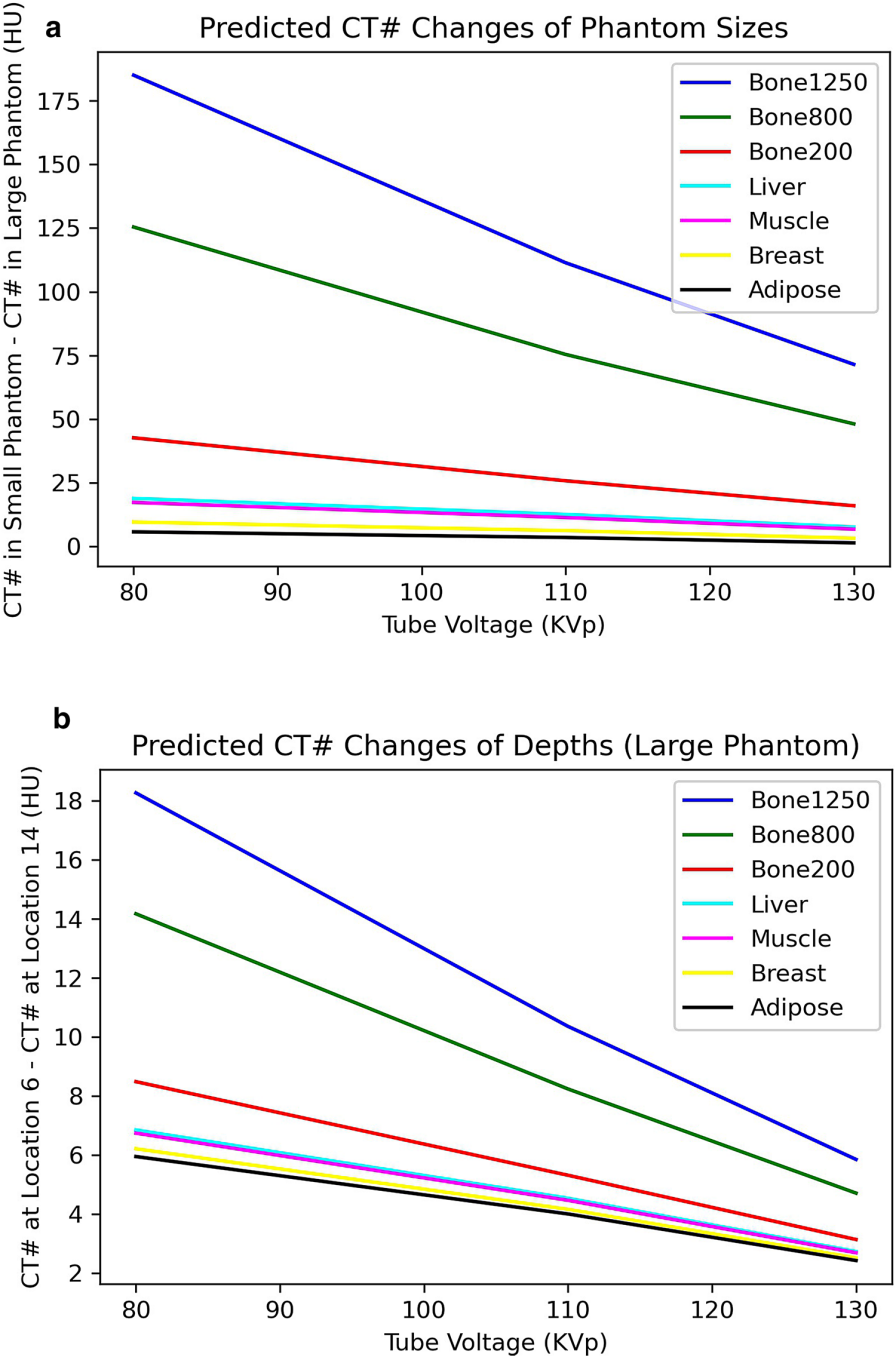
Predicted CT number changes employing the HU correction factor. (**a**) CT number changes at number 6 hole from small circular disk phantom to the large elliptical disk phantom; (**b**) CT number changes at from number 6 hole to number 14 hole within the same large elliptical disk phantom.

In order to use Eq. (17) to correct CT numbers or predict the measured CT number changes, information of the system’s calibration is required. Equation (16) assumes CT systems are calibrated at zero sized phantom (using ideal attenuation coefficient of water from NIST^15^ in Eq. (16)). As shown above, the Siemens CT scanner employed in this study can be assumed to be calibrated with an phantom size of averaged 30 cm in diameter (Fig. 5) at the tube voltage of 110 keV (Fig. 6b). An effective energy ${V}_{e}$ should be used in Eq. (17) because polychromatic X-rays are used in medical CT systems. The only parameter for Eq. (17) is $\varepsilon$=0.057, which is a middle value between 0.034 for body size and 0.068 for depth from the entrance surface^10^. For a specific CT scanner, parameter $\varepsilon$ may be adjusted because different beam filtrations are employed in different imaging systems and the effective energy (voltage) of the polychromatic X-rays is system dependent. For the Siemens CT scanner employed in this study, a value of $\varepsilon$ =0.045 was employed and effective energies of 58, 64 and 72 keV were used for tube voltages of 80, 110 and 130 keV^1,12^. Figures 7 shows the predicted CT number changes for both the size and depths effects.

## Discussion

The predicted CT number changes of Fig. 7 are in general agreement with experimentally measured changes of CT numbers in Fig. 6, in particular, the size effect on the CT number variations (Figs. 6a and 7a). The size effect has much larger impact on the CT numbers than that of the depth effect. For the depth effect or beam hardening, the changes of CT numbers are dependent on the manufacturer’s beam hardening correction algorithm within a specific CT system (Fig. 6b). A very different depth dependent CT number variation to Fig. 6b may be found from a different CT system because the beam hardening correction algorithms are CT system dependent. In contrast, the size effect on CT number variation is expected to be similar (independent on the beam hardening correction algorithm), assuming clinical CT systems are calibrated using an adult sized body phantom. The measured CT number changes from the body size effect shown in Fig. 6a is in general agreement with fig. 11a of Goodsitt et al.^3^ given body size differences between these two studies.

It is worth noting that image reconstruction algorithms also have a significant impact on CT numbers that CT values of an object may be influenced by the presence of other objects within the field of view^1^. The large sized objects employed by both Goodsitt et al.^3^ and this work were created by adding either a soft tissue or water rings to the small sized objects. Very different CT number changes can be found if the water balloon filled holes in our experiments were filled by inserts of tissue materials other than water or water equivalent soft tissues. Our correction scheme is therefore effective on correcting CT number variations caused by factors other than image reconstruction algorithms such as body size^3^, off-centre patient positioning^2^, tissue material identification^16^ and size effect on stopping power ratio in radiotherapy^17^.

The depth dependent effective attenuation of water was derived from the first principles Boltzmann transport equation. It provides a possible physical explanation on why children receive a higher dose than that of adults under the same CT imaging conditions^18^. The Boltzmann equation is widely used in neutron and electron transport studies but rarely applied in X-ray CT imaging^7^, except in Monte Carlo simulation^13^. Rayleigh scattering is relatively a small component in the total attenuation cross section of water^15,19^. This work demonstrated the important contribution of water’s Rayleigh scattering in clinical X-ray imaging^19,20^. It is surprising that a single system dependent variable $\varepsilon$ can be used to correct for both depth and body size effects with very different impacts on the variations of CT numbers. The main limitations of this work are that data were acquired from one CT system and CT number changes were measured from two phantom sizes. Further work is required to test this body size and tube voltage dependent correction scheme on different CT imaging systems employing various different body sizes.

## Conclusions

A body size and tube voltage dependent correction scheme is established for correcting CT numbers in Hounsfield Unit. Rayleigh scattering of water is the contributing factor for both body size and depth effects on the variations of CT numbers. Further work is required to test this body size and tube voltage dependent correction scheme on different CT imaging systems employing various body sizes.

## References

[1] Hsieh J (2015). Computed Tomography: Principles, Design Artefacts and Recent Advances.

[2] Szczykutowicz TP, DuPlissis A, Pickhardt PJ (2017). Variation in CT number and image noise uniformity according to patient positioning in MDCT. AJR.

[3] Goodsitt MM, Christodoulou G, Larson SC (2011). Accuracies of the synthesized monochromatic CT numbers and effective atomic numbers obtained with a rapid kVp switching dual energy CT scanner. Med. Phys..

[4] Schneider U, Pedroni E, Lomax A (1996). The calibration of CT Hounsfield units for radiotherapy treatment planning. Phys. Med. Biol..

[5] ICRU (2012). Radiation dose and image quality assessment in computed tomography. J ICRU.

[6] Gabbai M, Leichter I, Mahgerefteh S, Sosna J (2015). Spectral material characterization with dual-energy CT: Comparison of commercial and investigative technologies in phantoms. Acta Radiol..

[7] Fernandez, J. E. & Molinari, V.G. X-ray photon spectroscopy calculations in *Advances in Nuclear Science and Technology 22* (ed. Lewins J. & Becker M.) 45–104 (Plenum Press, 1991).

[8] Fernandez JE, Scot V, Di Giulio E, Verardi L (2011). Angular distribution of scattering intensities with the SAP code. X-Ray Spectrom..

[9] Berger, M.J., Hubbell, J.H., Seltzer, S.M., Chang, J., Coursey, J.S., Sukumar, R., Zuker, D.S., Olsen, K. XCOM: photon cross sections database. https://www.nist.gov/pml/xcom-photon-cross-sections-database. Accessed November 18 2018.

[10] Zheng X, Nardi L, Murray M (2017). Size effect on dose output in phantoms of x-ray tubes in medical X-ray imaging. Biomed. Phys. Eng. Express.

[11] Zheng X (2018). Body size and tube voltage dependent guiding equations for optimal selection of image acquisition parameters in clinical X-ray imaging. Radiol. Phys. Technol..

[12] McCullough EC (1975). Photon attenuation in computed tomography. Med. Phys..

[13] Salvat, F. PENELOPE-2014: A code system for Monte Carlo simulation of electron and photon transport. (Nuclear Energy Agency, Organisation for Economic Co-operation and Development, 2015)

[14] ImageJ software at https://imagej.nih.gov/ij/.

[15] NIST (n. d.). X-ray mass attenuation coefficients – water, liquid. https://physics.nist.gov/PhysRefData/XrayMassCoef/ComTab/water.html. Accessed November 18 2018.

[16] Taguchi K, Funama Y, Zhang M, Fishman EK, Geschwind JH (2009). Quantitative measurement of iodine concentration in the liver using abdomen C-arm computed tomography. Acad. Radiol..

[17] Inaniwa T, Tashima H, Kanematsu N (2018). Optimum size of a calibration phantom for x-ray CT to convert the Hounsfield units to stopping power ratio in charged particle therapy treatment planning. J. Radiat. Res..

[18] AAPM Task Group 204. Size specific dose estimates (SSED) in pediatric and adult body CT examinations. (American Association of Physicists in Medicine, 2011).

[19] John PC, Yaffe MJ (1983). Coherent scatter in diagnostic radiology. Med. Phys..

[20] Zhou A, White GL, Davidson R (2018). Validation of a Monte Carlo code system for grid evaluation with interference effect on Rayleigh scattering. Phys Med Biol.

